# Phytochemical constituents, antioxidant and antibacterial activities of *Plectocephalus varians (*A. Rich.) C. Jeffrey ex Cufod root extracts

**DOI:** 10.1186/s12906-023-03919-8

**Published:** 2023-04-28

**Authors:** Melaku Birhane Gashaye, Yihenew Simegniew Birhan

**Affiliations:** Department of Chemistry, Natural and Computational Sciences College, Debre Markos University, P.O. Box 269, Debre Markos, Ethiopia

**Keywords:** *P. varians*, Phytochemicals screening, Antibacterial activity, Antioxidant activity

## Abstract

Plants have been used to treat diverse types of diseases in different cultural groups around the globe. In this regard, the root of *Plectocephalus varians* (*P. varians*) is claimed to have a beneficiary effect in treating cancer and hemorrhoids in Ethiopia. Hence, this study aimed at the phytochemical investigation, antioxidant, and antibacterial activities of n-hexane, acetone, and methanolic extracts of *P. varians* root. The different crude extracts of *P. varians* were obtained through maceration technique. The total phenolic content (TPC) and total flavonoid contents (TFC) of the extracts were estimated using Folin-Ciocalteu Reagent (FCR) and aluminum chloride colorimetric assays, respectively. The antioxidant activities of the extracts were determined by 2,2-diphenyl-1-picrylhydrazyl (DPPH) free radical scavenging and ferric reducing antioxidant power (FRAP) assays. The antibacterial activities of the extracts were assessed by using disc diffusion method. The results echoed the presence of alkaloids, saponins, flavonoids, steroids, phenols, tannins, anthraquinones, terpenoids, polyphenols, and glycosides in the root of *P. varians*. The methanolic root extract (MRE) had the highest TPC (107.18 mg GAE/g) and TFC (120.194 mg QE/g) followed by acetone root extract (ARE) (TPC = 98.68 mg GAE/g; TFC = 64.038 mg QE/g) and n-hexane root extract (HRE) (TPC = 12.39 mg GAE/g; TFC = 9.917 mg QE/g). The DPPH radical scavenging and FRAP assays demonstrated the antioxidant effects of HRE (IC_50_ = 681.75 ppm; EC_50_ = 60.65 ppm), ARE (IC_50_ = 165.73 ppm; EC_50_ = 51.67 ppm) and MRE (IC_50_ = 132.06 ppm; EC_50_ = 30.97 ppm) of *P. varians*. Furthermore, the root fractions elicited pronounced dose-dependent growth inhibition against *Staphylococcus aureus*, *Streptococci pyogenes*, *Escherichia coli*, and *Klebsiella pneumoniae* with mean zone of inhibition (MZI) ranging from 11 ± 0.38 to 20 ± 0.04 mm at 800 ppm. Overall, the present study provides ethnopharmacological evidence suggesting the medicinal importance of *P. varians*. The results also call for further bioassay-guided phytochemical screening and in vitro and/or in vivo bioactivity testing.

## Introduction

Plants have been used as a source of medicine in traditional healthcare systems worldwide since immemorial [1]. Approximately 80% of the primary healthcare demands of low- and middle-income countries to treat different diseases primarily rely on traditional medicines [2]. In Ethiopia, approximately 80% of human and 90% of livestock populations depend on traditional medicines to meet their healthcare necessities [3] and more than 95% of the traditional preparations are acquired from plants [4]. The indiscriminate use of medicinal plants (MPs) for the management of different diseases in Ethiopia may be attributed to the rich biodiversity endowed with more than 6000 plant species [5], the existence of pluralities (language, culture, beliefs, etc.) which nurture the predominant indigenous knowledge or practices [6], and the abundance of different phytochemicals responsible for amelioration or attenuation of the underlying mechanisms implicated in the pathogenesis and progress of physical and mental disorders [7]. Traditional healthcare practitioners (THPs) prefer different organs of MPs alone or in combination for the formulation of recipes that can be applied through various routes of administration (ROA). In addition, THPs employ diverse techniques to extract the active constituents of MPs and use different types additives in the preparation of efficient and affordable herbal remedies characterized by lower side effects and better patient compliance [5, 8, 9].

In recent years, a considerable attention is given to the ethnobotanical study of MPs that mainly devoted to the collection, documentation and analysis of different aspects of plant-based traditional knowledge and practices pertaining to indigenous communities across the world including Ethiopia [10–12]. Most importantly, substantive efforts are being made to scientifically prove the biological activities of herbal formulations prescribed by traditional healers through in vitro and in vivo experiments: to assess the bioactivity and feasibility of herbal formulations in the clinical setting and to identify the pharmacologically active constituents that may potentially serve as a lead compound for drug development and applications [13]. In this regard, different plant-based crude extracts, solvent fractions, essential oils, isolated compounds, etc. are screened for their antibacterial and antioxidant activities among others [14–21]. For instance, the in vitro antioxidant and antibacterial activities of 80% methanolic root extracts of *Croton macrostachyus* was assessed [14] and the extracts showed promising DPPH radical scavenging activities (IC_50_ ranging from 3.5–6.4 mg AAE/g of extract) and bacteria growth inhibition against *Staphylococcus aureus, Staphylococcus pneumonia*, *Escherichia coli* and *Klebsiella pneumonia*, which was comparable with the reference drug, Gentamicin. In addition, Belay et al., [15] assessed the antioxidant and antibacterial activities of n-hexane, dichloromethane and ethyl acetate fractions of *Calpurnia aurea* leaves. The FRAP result of the n-hexane fraction (1.25 µg AAE/mg of extract) was comparable with the standard value (1.27 µg AAE/mg of extract). The dichloromethane and ethyl acetate fraction had a relatively lower reducing power of 0.93 and 1.13 µg AAE/mg of extract, respectively. Moreover, at 500 mg/well, the ethyl acetate fraction significantly inhibited growth of *Enterococcus faecalis* (18 mm) and *Salmonella typhi* (17.33 mm). Similar studies conducted on the flower extract of *Vernonia amygdalina* [16], aerial part of *Portulaca quadrifida* [17], rootbark of *Gnidia involucrate* [18], root extracts of *Cucumis prophetarum* [19], and leaf extracts of *Otostegia integrifolia* [20], etc. reiterated the antioxidant and antibacterial activities of the respective MPs, suggesting the indispensable role of plant-based medicines in combating bacterial infections and attenuating diseases caused by generation of free radicals.

*Plectocephalus varians* (A. Rich.) C. Jeffrey ex Cufod (known as “Etse-Yohannes” in Amharic) is one of the most common MP species grown in different parts of Ethiopia [22] which belongs to the Asteraceae family and is frequently employed in the treatment of human and livestock ailments in Ethiopia [23]. An ethnobotanical investigation conducted in Mecha District, West Gojjam Administrative Zone, Amhara Regional State, Ethiopia, documented the traditional healthcare importance of *P. varians* in the management of human diseases such as cancer and hemorrhoid [24]. The overproduction of free radicals (reactive oxygen and nitrogen species) and subsequent oxidative stress is among the various mechanisms associated with cancer development [25–27] and pathogenic bacteria strains may have a devastating role in sustaining hemorrhoid-related infections. Thus, the traditional healthcare practices pertinent to the use of *P. varians* for cancer and hemorrhoids might be related to their inherent antioxidant [28] and antibacterial activities. To the best of our knowledge, there is no report on the phytochemical profiles and bioactivities of *P. varians* root extracts. The present study aimed at qualitative and quantitative phytochemical investigations, antioxidant and antibacterial activities of HRE, ARE and MRE of *P. varians*.

## Materials and methods

### Chemicals and reagents

In the present study, analytical grade chemicals such as n-hexane, acetone, methanol, hydrochloric acid, sodium hydroxide, ferric chloride, chloroform, iodine, potassium iodide, concentrated sulfuric acid, benzene, ammonia, Gallic acid (GA), Folin-Ciocalteu Reagent (FCR), sodium carbonate, dimethyl sulfoxide (DMSO), 2,2-diphenyl-1-picrylhydrazyl (DPPH), quercetin, ascorbic acid (AA), trichloroacetic acid, potassium ferricyanide, monosodium hydrogen phosphate, and disodium hydrogen phosphate were purchased and used without any further purifications.

### Collection of plant materials

The plant material was collected from Yebokla *Kebele* (the smallest administrative unit in Ethiopia), Gozamen District, East Gojjam Zone Administrative Zone, Amhara Regional State, Ethiopia, which is 42 km far from Debre Markos town on November 2021. The collection of the plant material was performed in accordance with IUCN Policy Statement on Research Involving Species at Risk of Extinction (https://portals.iucn.org/library/efiles/documents/PP-003-En.pdf). The genus and species names of the collected plant materials were identified by a botanist from Debre Markos University, Department of Biology. The voucher specimen was deposited in Debre Markos University, Department of Chemistry. Then, the roots of *P. varians* were used in the extraction, qualitative and quantitative phytochemical analysis, antioxidant, and antibacterial activity testing.

### Extraction and phytochemical screening

The collected plant material was washed with tap water to remove dust, sediments, and parasites. The roots of *P. varians* were air-dried in Debre Markos University, Department of Chemistry laboratory room for 2 weeks and pulverized into powder with a mortar and pestle for subsequent extraction. The powdered roots of *P. varians* were successively macerated with n-hexane, acetone, and then with methanol in a conical flask [29]. Herein, 500 g root powder was soaked with each solvent (1:5 root powder solvent ratio). The mixture was constantly shaken and kept at room temperature (⁓25℃) for seven days. Then, the solutions were filtered through Whatman Number 1 filter paper. This process is repeated twice to maximize the extraction and fractionation of phytochemicals present in the root of *P. varians*. The filtrate was kept at 25℃ for a week (to trigger evaporation and significant decrement in the volume of the solvents) and the remaining solvents were removed by a rotary evaporator under vacuum to obtain the crude extracts. After drying, the mass of HRE, ARE and MRE was weighted and kept below 10℃ in a refrigerator until used for further experiments. The yields of HRE, ARE and MRE were computed according to the following formula:

$$\mathrm{The yield of crude extract }(\mathrm{\%}) = \frac{\mathrm{Weight of dry extracts}}{\mathrm{Weight dry plant material}}\times 100$$

Furthermore, the qualitative phytochemical analysis of HRE, ARE and MRE of *P. varians* was conducted by following the methods depicted in the literature [29–32]. The presence or absence of alkaloids, anthraquinones, flavonoids, glycosides, phenols, polyphenols, saponins, steroids, tannins, and terpenoids were assessed in the HRE, ARE and MRE of *P. varians*.

### Estimation of total phenolic content of extracts

The TPC of *P. varians* HRE, ARE, and MRE extracts was determined according to the Folin-Ciocalteu method as described in previous literature [33, 34]. Briefly, 1 mL (1 mg/mL) of HRE, ARE and MRE were separately added to 5 mL of 2N FCR. After 5 min, 4 mL 10% Na_2_CO_3_ solution was added to the mixture and mixed thoroughly and incubated at 37℃ for 45 min. Then, the absorbance of each extract solution was measured at 765 nm using a UV–vis spectrophotometer. Finally, the TPC was estimated from the standard calibration curve of GA in methanol (20, 40, 80, 150, 160, and 320 ppm). The experiments were carried out in triplicate. The TPC (in mgGAE/g of dry extract) in HRE, ARE and MRE was calculated as c(V/m), where c is the concentration of GA (in mg/mL) obtained from the calibration curve, V refers to the volume of extract (in mL), and m stands for the mass of extract (in g).

### Estimation of total flavonoid content of extracts

The TFC of *P. varians* HRE, ARE, and MRE extracts was quantitatively determined via the aluminum chloride colorimetric assay as described in previous literature [14] with slight modifications. In this method, quercetin was used as standard and the TFC was estimated as mg of quercetin equivalents/100 g of dry samples. In brief, 1 mL of standard quercetin solutions and each HRE, ARE and MRE solution (20, 40, 80, 150, 160 and 320 ppm) was transferred into 25 mL volumetric flask and 4 mL of distilled water and 0.3 mL of NaNO_2_ were added to each flask. After 5 min of incubation, 0.3 mL of 10% aluminum chloride followed by 2 mL of 1 M NaOH (6^th^ min) was added. Then, the absorbance of the standard, extract solutions and the blank (distilled water) were recorded at 510 nm using UV–vis spectrophotometer. The experiments were carried out in triplicate and the results are presented as mg QE/g dry weight of the HRE, ARE and MRE. Finally, the TFC was computed from the regression equation derived from the quercetin standard calibration curve.

### Antioxidant activity study

#### DPPH radical scavenging assay

The antioxidant activity of the HRE, ARE and MRE of *P. varians* was investigated by employing the DPPH free radical assay as mentioned in previous reports [35] with slight modifications. In brief, 10 mg of each HRE, ARE and MRE of *P. varians* sample was dissolved in 25 mL of methanol to serve as stock solutions. From the stock solutions, 25, 50, 100, 200, and 400 ppm working solutions were prepared for HRE, ARE and MRE. Likewise, different working concentrations of AA solutions were prepared from the stock solution as references. Then, 3 mL of DPPH with 3 mL of methanol was used as a negative control. To determine the radical scavenging activity of each extract, 3 mL of DPPH solution (1 M) was mixed with a 4 mL sample of each extract and the standard solution. After incubation for 30 min at 37℃, their absorbance was measured at 517 nm. All tests were performed in triplicate. The percent radical scavenging activity of the samples was calculated according to the following formula:

$$\text{DPPH radical scavenging activity }(\text{\%}) = \frac{\text{Absorbance of control}-\text{Absorbance of test sample}}{\text{ absorbance of control}} \times 100$$

#### Ferric reducing antioxidant power assay

The FRAP of HRE, ARE and MRE of *P. varians* was determined by following previous reports [26] with slight modification. Briefly, 2.5 mL of each extract sample (25, 50, 100, 200, and 400 ppm), 2.5 mL of 0.2 M sodium phosphate buffer (pH = 6.6) and 2.5 mL of 1% potassium ferricyanide [K_3_Fe(CN)_6_] solution was added. The reaction mixture was vortexed well and then incubated at 50℃ for 20 min. Then 2.5 mL of trichloroacetic acid was added and the solution was centrifuged for 10 min at 3000 rpm. The supernatant (2.5 mL) was mixed with 2.5 mL of distilled water and 0.5 mL of 0.1% ferric chloride solution. Moreover, 2.5 mL of distilled water was used as a blank solution. The absorbance of the colored solutions was recorded at 700 nm against the blank using a UV–vis spectrophotometer. Herein, AA was used as a reference standard and the reducing power of each sample was compared with the reference standard solution (prepared in the same way as the extract samples). The percentage inhibition of FRAP was calculated as follows:

$$\mathrm{FRAP }(\mathrm{\%}) = \frac{\mathrm{Absorbance of sample}-\mathrm{Absorbance of blank}}{\mathrm{ absorbance of sample}} \times 100$$

### Antibacterial activity study

The HRE, ARE and MRE were evaluated for their antibacterial activity against Gram-positive and Gram-negative bacteria strains. Four bacteria strains, *Staphylococcus aureus* (ATCC25923), *Streptococci pyogenes* (ATCC19615), *Escherichia coli* (ATCC25922), and *Klebsiella pneumoniae* (ATCC700603) were used for antibacterial screening. The antibacterial activity screening was carried out by disc diffusion method on Mueller–Hinton agar [36]. A stock solution with different concentrations (50, 100, 200, 400, and 800 ppm) of each sample (HRE, ARE and MRE), Gentamycin, and DMSO were added to the incubation plate by using a 6 mm filter paper with the help of a micropipette. The discs belonging to HRE, ARE and MRE, standards as well as blank discs were placed on the surface of the Petri dish equidistantly. The plate was incubated at 37℃ for 24 h. Then, the antibacterial activity was computed by measuring the diameter of the inhibition zone (in mm) against each test organism treated at different concentrations and recorded if the zone of inhibition was greater than 6 mm. The measurement was performed in triplicate and the results were expressed as mean ± standard deviation.

### Statistical analysis

Results were presented as mean ± standard deviation (SD). One-way analysis of variance (ANOVA) was used to assess the presence of significant variations among test groups in the antimicrobial activity screening and *p* values less than 0.05 were considered as statistically significant.

## Results and discussion

### Extraction yield and phytochemical screening

The extraction of phytochemicals from different plant organs entirely depend on the type or polarity of solvents [37]. The air-dried root of *P. varians* was macerated in n-hexane, acetone, and methanol with increasing polarity for sequential extraction of phytoconstituents based on the principle of “like dissolve-like”. The result showed that the percentage yield of HRE, ARE and MRE was found to be 1.15, 2.25, and 1.82%, respectively (Table 1). The solvent with medium polarity, acetone, experienced a highest extraction yield compared to the less polar n-hexane and more the polar methanol solvents, signifying the abundance of phytochemicals with medium polarity in the root of *P. varians*. On the contrary, acetone resulted in the least yield (1.05%) of whole plant *Spilanthes mauritiana* extract as compared to methanol (2.95%) and n-hexane (2.8%), suggesting the yield of extraction entirely depend on the polarity of the phytochemicals present in the plant organs [37]. The qualitative phytochemicals screening tests revealed the presence of alkaloids, glycosides, steroids, terpenoids, and polyphenols in HRE, ARE and MRE regardless of the variation in concentrations (Table 2). Unlike the MRE which has shown a positive test for all secondary metabolites, ARE and HRE were devoid of some phytochemicals such as phenols, tannins, and anthraquinone (in n-hexane), flavonoids (in acetone) and saponins (in both n-hexane and acetone). Overall, ARE and MRE possessed the majority of phytochemicals asserting the abundance of relatively polar secondary metabolites in the root of *P. varians*. Similar reports reiterated that more polar extraction solvents picked up the phytochemical constituents more efficiently than less polar solvents [38–40].

**Table 1:** The percentage yields of *P. varians* root crude extracts

Extract	Weight of extract (in g)	% Yield
n-hexane	5.76	1.15
Acetone	11.26	2.25
Methanol	9.10	1.82

**Table 2:** Qualitative phytochemical analysis of *P. varians* root crude extracts

Phytochemicals	Reagents	Crude extracts		
		**n-Hexane**	**Acetone**	**Methanol**
Alkaloids	Wagner’s test	+ +	+	+ + +
Anthraquinones	Borntrager’s test	_	+	+ + +
Flavonoids	H_2_SO_4_ test	+ +	_	+ + +
Glycosides	Keller- Killiani test	+ +	+	+
Phenols	Ferric Chloride test	_	+ +	+ + +
Polyphenols	Lead Acetate test	+	+	+ +
Saponins	Foam test	_	_	+ +
Steroids	Salkowski’s test	+	+ + +	+ +
Tannins	Ferric Chloride test	_	+ + +	+ + +
Terpenoids	Salkowski’s test	+ + +	+ +	+ +

(-), not detected (ND); ( +), present; (+ +), moderately present and (+ + +), highly present

### Total phenolic contents of *P. varians* root extracts

The TPC of *P. varians* root solvent fractions were determined by using the FCR. In the FCR assay, as the polarity of the solvent increases the yellow-colored Mo^6+^ is changed to a lower oxidation state (Mo^+4^, Mo^+5^) with blue color. The reaction between the hydroxyl group of phenols with FCR to form a phosphomolybdate complex that can absorb UV light at 765 nm, is responsible for the appearance of the blue color [41]. The intensity of the blue color is directly associated with the concentration of phenolic compounds in the sample. In this study, GA equivalents per 100 g of dry weight of the sample (mg of GAE/100 g) served as a standard to compare the TPC of HRE, ARE and MRE as shown in Table 3. The result revealed that the MRE had highest level of phenolic content (107.182 mg GAE/100 g) while the HRE of the *P. varians* possessed the least phenolic content (12.394 mg GAE/100 g). As expected, the highest phenolic content was recorded in the more polar solvent extract [39] and vice versa. The result suggested that the extraction of phenolic compounds is entirely dependent on the polarity of the extraction solvent used. In line with this, the MRE of *P. varians* experienced a relatively more intense blue color compared to the HRE with low intensity. Phenolic compounds are important class of plant metabolites responsible for scavenging free radicals due to the presence of one or more hydroxyl groups in their structure. It is worthy of mentioning that nonphenolic phytoconstituents are also implicated in the antioxidant activities of extracts [42].

**Table 3:** Total phenolic and flavonoid contents of *P. varians* root extracts

Crude extracts	TPC (mg GAE/g extracts)	TFC (mg QE/g extracts)
n-Hexane	12.394	9.917
Acetone	98.697	64.038
Methanol	107.182	120.194

### Total flavonoid contents of *P. varians* root extracts

The TFC of *P. varians* HRE, ARE, and MRE extracts was quantitatively determined via the aluminum chloride colorimetric assay. In this study, quercetin equivalents per 100 g of dry weight of the sample (mg of QE/100 g) served as a standard to compare the TFC of HRE, ARE and MRE as shown in Table 3. The result revealed that the MRE had highest level of flavonoid content (120.194 mg QE/100 g) while the HRE of the *P. varians* possessed the least flavonoid content (9.917 mg QE/100 g). While ARE had optimum flavonoid content of 64.038 mg QE/100 g. Like the TPC, the highest TFC was recorded on the MRE. This might be attributed to the magnitude in the polarity of the extraction solvent which is vital to abstract polar phytoconstituents from *P. varians* roots such as flavonoids based on the principle of “like dissolve like”. Flavonoids are another class of relatively polar phytochemicals that contain hydroxyl groups implicated in absorbing and neutralizing free radicals, quenching singlet and triplet oxygen, or decomposing peroxides [40] and thereby ameliorate the undesirable effects of free radicals such as oxidative stress in cells.

### Determination of antioxidant capacity

#### DPPH radical scavenging assay

**Fig. 1:**
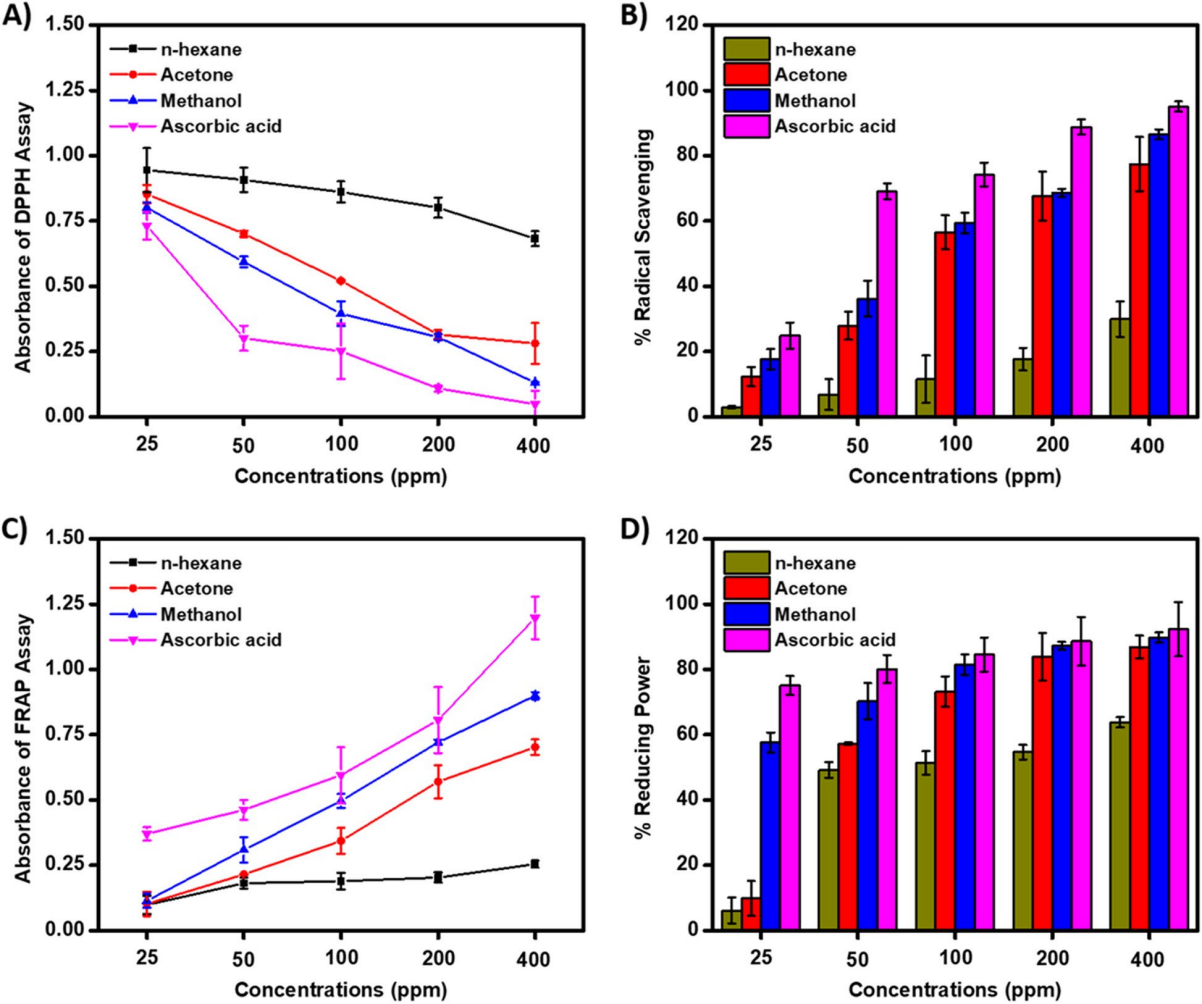
Absorbance of DPPH assay (**A**), radical scavenging activity (**B**), absorbance of FRAP assay (**C**) and percent reducing power (**D**) of root extracts of *P. varians* and AA at different concentrations

The DPPH radical scavenging assay is a short and efficient method extensively employed to determine the antioxidant capacity of plant extracts [39]. The antioxidant activity of HRE, ARE and MRE of *P. varians* was assessed by using DPPH radical scavenging assay. DPPH is a stable free radical that accepts an electron or hydrogen radical from stable 2,2-diphenyl-l-picryl hydrazine, consequently, the scavenging assay undergoes a change in color from purple to yellow. In the present study, as the concentration of samples and standard AA increased, the DPPH electron scavenging activity increased (Fig. 1A), which is accompanied by the appearance of a characteristic yellow color. The free radical scavenging effect of different root extracts of *P. varians* on the DPPH radical decreases from the less polar solvent n-hexane (IC_50_ = 681.75 ppm) to the more polar solvent methanol (IC_50_ = 132.06 ppm). The highest percentage inhibition (Fig. 1B) for the MRE as compared to ARE and HRE (Table 4) could be attributed to the presence of high concentration of phenolic compounds (such as polyphenols, tocopherols, etc.) capable of donating hydrogen atoms. In general, the concentration of phenolic compounds plays a paramount role in the antioxidant or scavenging activity of different solvent extracts: the higher the phenolic content, the better will be the free radical scavenging or inhibition activity [39]. Similar trends were observed in the DPPH antioxidant activity of methanolic and n-hexane stembark extracts of *Beilschmiedia roxburghiana*, where the methanolic extract showed superior antioxidant activity as compared to the n-hexane extract. In this study, the correlation of TPC and TFC of different solvent extracts with DPPH radical scavenging capacity was assessed and the result revealed a good correlation between DPPH radical scavenging activity with TPC (R^2^ = 0.998) and TFC (R^2^ = 0.788) was observed. The result is consistent with previous reports about the relative abundance of TPC/TFC and subsequent DPPH scavenging potential of MPs proved to have antioxidant activities [17, 26]. The results of the present study indicated that the extracts showed radical scavenging activity due to their electron transfer or hydrogen donating ability because the TPC and TFC in plants are proportional to the radical scavenging activity [39].

**Table 4:** The IC_50_ and EC_50_ values of *P. varians* root extracts against DPPH and FRAP assays

Crude Extract	IC_50_ (ppm)	EC_50_ (ppm)
n-Hexane	681.75	60.65
Acetone	165.73	51.67
Methanol	132.06	30.97
Ascorbic Acid	5.88	0.10

IC_50_ and EC_50_ refers to the concentration of the root extract that inhibit 50% of the DPPH and 50% of the FRAP capacity, respectively

#### Ferric reducing antioxidant power assay

In addition to the DPPH radical scavenging assay, the antioxidant activity of HRE, ARE and MRE of *P. varians* was assessed by using the FRAP assay. The FRAP assay undergoes reduction from ferricyanide (Fe^3+^) to ferrocyanide (Fe^2+^) which is accompanied by the appearance of blue color when subjected to antioxidant species which can donate an electron [26]. The intensity of the characteristic color, which ranges from blue to Prussian blue, depends on the concentration of the sample containing the antioxidant species. This phenomenon may be attributed to an increase in electron donor moieties that can reduce ferricyanide (Fe^3+^) to ferrocyanide (Fe^2+^). The amount of Fe^2+^ formed in the process can be determined from the UV–vis absorption of ferrocyanide at 700 nm. In the present study, the MRE of *P. varians* showed significantly (*p* < 0.05) higher absorbance (Fig. 1C) and FRAP reducing power (Fig. 1D) as compared with HRE. In addition, ARE also showed higher FRAP values when compared with HRE. The higher FRAP reducing power of MRE and ARE may be associated with the presence of polar phytoconstituents such as polyphenolic compounds and flavonoids capable of reducing ferricyanide (Fe^3+^) to ferrocyanide (Fe^2+^). Since the reducing capacity of an extract is an important indicator of its antioxidant capacity [23], the findings provided pieces of evidence for the antioxidant potential of the *P. varians* root, especially MRE (EC_50_ = 30.97 ppm) and ARE (EC_50_ = 51.67 ppm) (Table 4). Moreover, the correlation of TPC and TFC of different solvent extracts with FRAP assay was assessed and the findings revealed a good correlation between FRAP percent inhibition with TPC (R^2^ = 0.998) and TFC (R^2^ = 0.618) was recorded. Other reports also suggested the remarkable potency of methanolic extracts to terminate the free radical chain reactions by converting them into stable nonreactive species through donating electrons to reactive free radicals [43]. Overall, the DPPH radical scavenging and FRAP assay results suggested the potential of *P. varians* root in the amelioration of diseases triggered by the generation and accumulation of reactive oxygen species in the body [26].

### Antibacterial Activity of *P. varians* root extracts

**Fig. 2:**
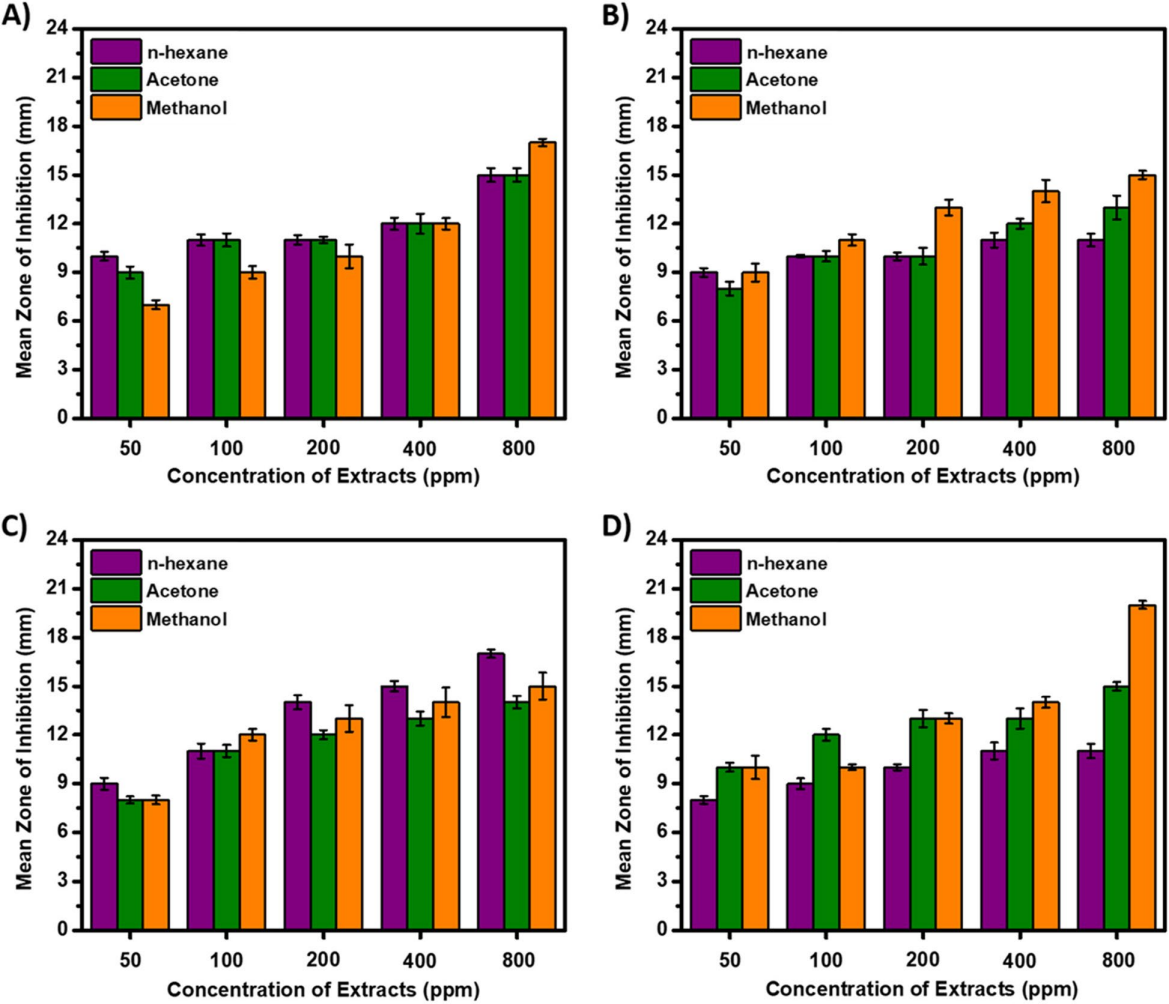
The antibacterial activities of *P. varians* root extracts against *Staphylococcus aureus* (**A**), *Streptococcus pyogenes* (**B**), *Escherichia coli* (**C**), and *Klebsiella pneumoniae* (**D**)

There is ample evidence for the antibacterial activities of plant extracts and their phytoconstituents against various pathogenic bacteria strains of Gram-positive and Gram-negative nature [19]. The antibacterial activities of HRE, ARE and MRE were assayed against two Gram-positive and two Gram-negative bacteria strains relative to the standard antibacterial drug Gentamicin by using the disc diffusion method [36]. The zones of inhibition (in mm) for the controls and *P. varians* root extracts against *Staphylococcus aureus* (Fig. 2A), *Streptococcus pyogenes* (Fig. 2B), *Escherichia coli* (Fig. 2C) and *Klebsiella pneumoniae* (Fig. 2D) were measured and analyzed. Herein, the standard drug (Gentamycin, 10 ppm) experienced the highest mean zone of inhibition (MZI) in all test bacteria strains (27 ± 0.26 mm) as compared to DMSO (negative control, 6 ppm) and the different root extracts of *P. varians* (ranging from 50 to 800 ppm). All the *P. varians* root extracts elicited diverse bacterial growth inhibition effect in the tested strains, albeit, their MZI increased in a dose-dependent manner regardless of the type of bacteria strains. At the maximum dose (800 ppm), the HRE of *P. varians* showed maximum MZI against *Escherichia coli* (17 ± 0.15 mm) followed by *Staphylococcus aureus* (15 ± 0.43 mm). HRE had comparable growth inhibition effect against *Streptococcus pyogenes* (11 ± 0.38 mm) and *Klebsiella pneumoniae* (11 ± 0.44 mm) at the same dose. Similarly, the ARE elicited comparable MZI against *Staphylococcus aureus* (15 ± 0.62 mm) and *Klebsiella pneumoniae* (15 ± 0.26 mm). Mild growth inhibition effect of the ARE against *Streptococcus pyogenes* (13 ± 0.73 mm) and *Escherichia coli* (14 ± 0.39 mm) was detected in the disc diffusion assay. Interestingly, the MRE exhibited broad-spectrum antibacterial activity against all the tested pathogens with MZI ranging from 15 ± 0.25 to 20 ± 0.04 mm. Briefly, the MRE recorded the highest MZI against *Klebsiella pneumoniae* (20 ± 0.04 mm) accompanied by *Staphylococcus aureus* (17 ± 0.12 mm). Moreover, MRE exhibited comparable antibacterial activity against *Streptococcus pyogenes* (15 ± 0.25 mm) and *Escherichia coli* (15 ± 0.83 mm) at a dose of 800 ppm. Overall, relatively highest antibacterial activities were recorded by the MRE against *Klebsiella pneumoniae* (20 ± 0.04 mm) and *Staphylococcus aureus* (17 ± 0.12 mm); and ARE on *Escherichia coli* (17 ± 0.15 mm). Consistent with this, Yitayeh et al., [40] also reported the superior antibacterial activity of the methanolic *Solanecio gigas* stembark extract as compared to the less polar ethyl acetate and chloroform extracts on the same bacteria strains. The significant and better antibacterial activity of the MRE of *P. varians* might be attributed to the presence of high concentrations of flavonoids, alkaloids, phenols, terpenoids, tannins, etc. with profound antibacterial effects [44, 45]. Hydroxyl moieties of secondary metabolites such as phenolic, polyphenols, flavonoids, tannins, etc. are implicated in the toxicity of microorganisms through disruption of cell membranes and spontaneous outflow of intracellular components that can potentially culminate in the death of pathogenic bacteria strains [21, 42]. The pronounced antibacterial activity of the MRE is consistent with the TPC and TFC as reported elsewhere [40].

## Conclusions

In the present study, the qualitative phytochemical screening of HRE, ARE and MRE revealed the presence of notable secondary metabolites in the root of *P. varians*. The different root extracts of *P. varians* elicited antioxidant activities in DPPH radical scavenging and FRAP assays. In addition, a strong correlation between the TPC and TFC with DPPH scavenging activity and FRAP reducing potential was observed. Moreover, the FRAP assay also revealed the desirable antioxidant activities of *P. varians* root extracts, especially MRE and ARE, which might be attributed to the abundance of phenolic compounds and flavonoids in the extracts. Moreover, the HRE, ARE and MRE of *P. varians* elicited growth significant inhibition against Gram-positive and Gram-negative bacteria strains. Overall, the result proved the existence of strong association between the indigenous knowledge of THPs and *P. varians* as far as its beneficial effect in treating cancer and hemorrhoids. The preliminary findings call for further rigorous investigations including but not limited to HPLC–MS analysis, bioassay-guided isolation and characterization of bioactive compounds and in vivo acute toxicity studies in different animal models.

## Data Availability

The datasets used and/or analyzed in the current study can be shared from the corresponding author on reasonable request.

## Abbreviations

ATCC: American Type Culture Collection
ARE: Acetone root extract
HRE: Hexane root extract
MRE: Methanol root extract
DPPH: 2,2-Diphenyl-1-picrylhydrazyl
DMSO: Dimethylsulfoxide
FRAP: Ferric reducing antioxidant power
FCR: Folin-Ciocalteu Reagent
MZI: Mean zone of inhibition
MPs: Medicinal plants
P. varians: Plectocephalus varians
ROA: Routes of administration
TFC: Total flavonoid content
TPC: Total phenolic content
THPs: Traditional healthcare practitioners
